# Identification of hub genes within the CCL18 signaling pathway in hepatocellular carcinoma through bioinformatics analysis

**DOI:** 10.3389/fonc.2024.1371990

**Published:** 2024-03-06

**Authors:** Jinlei Mao, Yuhang Tao, Keke Wang, Hanru Sun, Manqi Zhang, Liang Jin, Yi Pan

**Affiliations:** State Key Laboratory of Natural Medicines, Jiangsu Key Laboratory of Druggability of Biopharmaceuticals, School of Life Science and Technology, China Pharmaceutical University, Nanjing, Jiangsu, China

**Keywords:** hepatocellular carcinoma, CCL18, tumor microenvironment, prognostic, diagnosis

## Abstract

**Introduction:**

Hepatocellular carcinoma (HCC) is an aggressive malignancy, and CCL18, a marker of M2 macrophage activation, is often associated with tumor immune suppression. However, the role of CCL18 and its signaling pathway in HCC is still limited. Our study focuses on investigating the prognostic impact of CCL18 and its signaling pathway in HCC patients and biological functions *in vitro*.

**Methods:**

HCC-related RNA-seq data were obtained from TCGA, ICGC, and GEO. The 6 hub genes with the highest correlation to prognosis were identified using univariate Cox and LASSO regression analysis. Multivariate Cox regression analysis was performed to assess their independent prognostic potential and a nomogram was constructed. *In vitro* experiments, including CCK8, EdU, RT-qPCR, western blot, and transwell assays, were conducted to investigate the biological effects of exogenous CCL18 and 6 hub genes. A core network of highly expressed proteins in the high-risk group of tumors was constructed. Immune cell infiltration was evaluated using the ESTIMATE and CIBERSORT packages. Finally, potential treatments were explored using the OncoPredict package and CAMP database.

**Results:**

We identified 6 survival-related genes (BMI1, CCR3, CDC25C, CFL1, LDHA, RAC1) within the CCL18 signaling pathway in HCC patients. A nomogram was constructed using the TCGA_LIHC cohort to predict patient survival probability. Exogenous CCL18, as well as overexpression of BMI1, CCR3, CDC25C, CFL1, LDHA, and RAC1, can promote proliferation, migration, invasion, stemness, and increased expression of PD-L1 protein in LM3 and MHCC-97H cell lines. In the high-risk group of patients from the TCGA_LIHC cohort, immune suppression was observed, with a strong correlation to 21 immune-related genes and suppressive immune cells.

**Conclusion:**

Exogenous CCL18 promotes LM3 and MHCC-97H cells proliferation, migration, invasion, stemness, and immune evasion. The high expression of BMI1, CCR3, CDC25C, CFL1, LDHA, and RAC1 can serve as a biomarkers for immune evasion in HCC.

## Introduction

Liver cancer is the sixth most common primary malignant tumor and the fourth leading cause of cancer-related death worldwide, with a five-year survival rate of 21%. Hepatocellular carcinoma (HCC) accounts for more than 90% of liver cancer cases (1, 2). Although hepatitis B virus (HBV), hepatitis C virus (HCV), and alcohol remain important risk factors, the prevalence of obesity and diabetes has made non-alcoholic fatty liver disease (NAFLD) or non-alcoholic steatohepatitis (NASH) a dominant risk factor for HCC (3).

HCC presents intricate molecular characteristics and various pathological subtypes in a more natural manner, and the recommended treatment strategy for patients with advanced HCC continues to be systemic therapy, utilizing first-line agents like Sorafenib and Lenvatinib (3, 4). In recent times, there has been a growing focus on immune checkpoint inhibitors (ICIs) for the treatment of HCC. The combination of Atezolizumab (anti-programmed death-ligand 1) and Bevacizumab (anti-vascular endothelial growth factor) has emerged as a new standard for patients with advanced HCC (5), offering a therapy that modulates the HCC microenvironment. However, it is important to note that this treatment is only effective in a minority of HCC patients (6). In this regard, further research is needed to better understand the tumor microenvironment in HCC. This will allow for the identification of biomarkers that can be used to develop personalized treatment strategies.

Tumor microenvironment (TME) is a complex ecosystem that encompasses diverse immune cells, including dendritic cells (DC), monocytes, macrophages, B cells, and T cells (7). The TME of HCC accelerates tumor cell proliferation, invasion, and metastasis by forming an immunosuppressive environment (8). Tumor-associated macrophages (TAMs) have a high proportion in HCC TMEs and contribute to angiogenesis, cancer cell progression, and treatment resistance (9, 10). Macrophages can be classified into two main types: M1 and M2. M1 macrophages are involved in the immune response against cancer cells and express the CD86 marker. On the other hand, M2 macrophages have immunosuppressive functions and express the CD163 and CD206 markers (11). Of note, Guo et al. demonstrated that there is a subgroup of M2 macrophages (CD68^+^ CD206^+^) with high expression of chemokine ligand 18 (CCL18) in the HCC microenvironment and may be involved in the HCC process (12).

CCL18 is a chemokine secreted by TAMs and serves as a biomarker for M2 macrophages. It has been shown to promote tumor cell proliferation and facilitate immune evasion, aiding in the progression of tumor growth (13, 14). Lin et al. reported that CCL18 can promote HCC cell migration and invasion (15). However, research on the immunosuppressive effects of CCL18 in HCC is relatively limited. In this study, we systematically investigated the impact of the CCL18 signaling pathway on the prognosis of HCC patients. Consequently, six hub genes associated with prognosis were determined through bioinformatics analysis. These genes are denoted as BMI1, CCR3, CDC25C, CFL1, LDHA, and RAC1. Subsequently, we validated the biological functions of exogenous CCL18 and these six genes in LM3 and MHCC-97H cells through experimental assays. Then, we conducted immune cell infiltration of high-risk group and verified the influence of exogenous CCL18 and the expression of hub genes on PD-L1 protein. Eventually, potential treatments were explored using computational tools. The aim of this research is to gain a better understanding of the mechanisms underlying the development of immunosuppressive malignant HCC associated with CCL18. Identifying relevant tumor biomarkers may serve as a reference for diagnostic and immunotherapy of HCC.

## Materials and methods

### Database selection and data acquisition

In this study, we acquired gene expression matrix (RNA-seq) and clinical information of HCC patients from The Cancer Genome Atlas (TCGA) (https://portal.gdc.cancer.gov/; TCGA_LIHC; 362 patient samples), International Cancer Genome Consortium (ICGC) (https://dcc.icgc.org/; ICGC_JP; 230 patient samples), and Gene Expression Omnibus (GEO) (GSE14520; 225 patient samples) (**Table 1**). The tumor samples for RNA-seq included 374 cases for TCGA_LIHC, 243 cases for ICGC_JP, and 225 cases for GSE14520. Accordingly, the adjacent normal samples of RNA-seq included 50 cases for TCGA_LIHC, 202 cases for ICGC_JP, and 220 cases for GSE14520. TCGA_LIHC dataset was used as the internal training cohort while ICGC_JP and GSE14520 datasets were used as the external testing cohorts. GSE14520 dataset was downloaded through “GEOquery” R package, and clinical data was acquired from the website. All TCGA_LIHC and ICGC_JP data, gene-expressed profile and clinical details were manually downloaded from the website.

**Table 1:** Clinical characteristics of the HCC patients in this research.

TCGA_LIHC			ICGC_JP			GSE14520		
Number of tumor patients 362			Number of tumor patients 230			Number of tumor patients 225		
**Age** **Gender** **T_stage** **N_stage** **M_stage** **Stage**	>=60 <60 Male Female T1 T2 T3 T4 TX Unknow N0 N1 NX Unknow M0 M1 MX stageI stageII stageIII stageIV unknow	198(54.25%) 164(45.75%) 245(67.12%) 117(32.88%) 178(49.17%) 90(24.86%) 78(21.55%) 13 1 2 247(68.23%) 4 110(30.39%) 1 260(71.82%) 3 99(27.35%) 168(83.46%) 83(22.92%) 83(22.92%) 4 24	**Age** **Gender** **Stage**	>=60 <60 Male Female stageI stageII stageIII stageIV	185(80.43%) 45(19.57%) 170(73.91%) 60(26.09%) 35(15.22%) 105(45.65%) 71(30.87%) 19(8.26%)	**Age** **Gender** **TNM_stage** **BCLC_stage** **CLIP_stage**	>=60 <60 unknow Male Female unknow stageI stageII stageIII unknow Stage_A Stage_B Stage_C unknow Stage_0 Stage_1 Stage_2 Stage_3 Stage_4 Stage_5 unknow	43(19.11%) 178(79.11%) 4 191(84.89%) 30(13.33%) 4 93(41.33%) 77(34.22%) 48(21.33%) 6 148(65.78%) 22(9.78%) 29(12.89%) 26 97(43.11%) 74(32.89%) 35(15.56%) 9 3 1 6

Meanwhile, 99 genes that are implicated in the CCL18 signaling pathway were obtained from WikiPathways (https://www.wikipathways.org/) (**Table S1**). Immune-related genes were obtained from the ImmPort database (https://www.immport.org/shared/home).

### Identification of hub genes

We utilized the “survival” and “survminer” R packages to perform univariate Cox regression analysis in the TCGA_LIHC training cohort with the aim of identifying genes that have a substantial impact on survival. Consequently, we observed significant variations in the expression of 39 genes. Subsequently, the “glmnet” R package was employed to conduct LASSO regression, enabling the selection of the most crucial variables from this gene set. As a result, a subset of 6 genes, specifically BMI1, CCR3, CDC25C, CFL1, LDHA, and RAC1, were identified as prominent hub genes.

### Construction of prediction model

The multivariate Cox regression analysis was employed using the 6 hub genes. The risk score for individual patients in TCGA_LIHC cohort was calculated using the following formula.


$$Risk\;score=\;\sum_{i=1}^{n}coefficient\left(i\right)\;\times \;gene\left(i\right)$$

In this formula, coefficients were acquired from multivariate Cox regression, where gene(i) represents mRNA expression. The patients in both the training and test cohorts were classified into two groups, namely the high- and low-risk groups, based on the median value of the risk score. Additionally, we examined whether the risk score independently served as a prognostic factor. The clinical characteristics within TCGA_LIHC cohort including age, gender, M, N, T stage, stage and risk score were analyzed through univariate and multivariate Cox regression analysis. Analogous analyses were performed using the ICGC_JP and GSE14520 cohorts.

A predictive nomogram was developed using the 6 hub genes to estimate the survival probability for individual HCC patients. The predictive capability of the nomogram was evaluated in both the training and test cohorts through the utilization of receiver operating characteristic (ROC) curves and calibration curves. The “rms”, “timeROC”, and “pROC” R packages were utilized for these assessments.

### Survival and pathway correlation analysis

To evaluate the influence of the six hub genes on the overall survival rate of TCGA_LIHC cohort, survival curves were generated using the Kaplan-Meier method from the “survival” R package, and the median value was taken as the best cut-off. The Kaplan-Meier Plotter (http://kmplot.com/analysis/) platform was employed to examine the association between the expression levels of prognostic genes and various clinical endpoints, including overall survival (OS), recurrence-free survival (RFS), progression-free survival (PFS), and disease-specific survival (DSS). Additionally, the genes encompassed within pathways were collected and subjected to analysis using the “GSVA” R package, with the parameter method = ‘ssgsea’ being specifically chosen. Subsequently, the correlation between the prognostic genes and the pathways was assessed using Spearman correlation analysis (16).

### Functional enrichment analysis and establishment of a PPI network

The “limma” R package was utilized to identify differentially expressed genes (DEGs) in the TCGA_LIHC cohort. This included comparisons between HCC tumor and normal samples, as well as between high- and low-risk groups (Log2 fold change > 1, *p* value< 0.05). A total of 308 genes were identified as the intersection between the genes exhibiting high expression in tumors and the genes within the high-risk score group. The findings were visually represented through the utilization of volcano and Venn diagrams using “ggvenn”, “tidyverse”, and “ggrepel” R packages, effectively illustrating the intersection of 308 genes exhibiting high expression in tumors and the genes within the high-risk score group.

The functional enrichment analysis of DEGs was systematically performed using the “clusterProfiler” and “org.Hs.eg.db” R packages, which facilitated Gene Ontology (GO) and Kyoto Encyclopedia of Genes and Genomes (KEGG) analyses for a comprehensive understanding of the biological functions involved. The protein-protein interaction (PPI) network was meticulously examined utilizing the STRING database (https://cn.string-db.org/), and a significant molecular cluster was identified through the application of MODE, a Cytoscape plugin (Cytoscape software version 3.9.1).

### Estimation of immune cell infiltration

The estimation of stromal, immune, and ESTIMATE scores was performed using the “ESTIMATE” R package, which provides a computational approach to calculate and quantify the stromal and immune components within the tumor microenvironment. Meanwhile, the “CIBERSORT” R package was utilized to estimate the impact of the risk score on the proportions of 22 immune cell subtypes in the TCGA_LIHC training cohort.

### Drug sensitivity analysis

The “OncoPredict” R package was utilized to predict drug sensitivity based on gene expression profiles. This approach enabled the calculation of drug sensitivity values for each sample, with lower values indicating higher efficacy of the drug. Comprehensive drug sensitivity analysis was conducted on all samples to determine the drug sensitivity values for commonly used tumor drugs.

CAMP (https://clue.io/) is an extensive database and analysis platform that offers valuable resources and tools to delve into and comprehend gene expression profiles and drug perturbations. In order to identify potential small-molecule drugs for the treatment of high-risk patients, we utilized the CAMP online database. Specifically, we selected downregulated genes from the pool of significant genes, as well as upregulated genes from the top 150 genes. By inputting this gene set into the CAMP database, we conducted a meticulous screening to identify promising small-molecule drugs capable of modulating the dysregulated gene expression patterns associated with high-risk patients.

### Cell culture and transfection

L02, Huh7, HepG2, LM3 and MHCC-97H cells were purchased from the Cell Bank of the Chinese Academy of Sciences (Shanghai, China). Cells were incubated in DMEM medium with 10% fetal bovine serum (FBS) and maintained in penicillin (100 IU/mL) and streptomycin (100 mg/mL) in 5% CO2 at 37°C. The plasmids encoding the BMI1, CCR3, CDC25C, CFL1, LDHA, and RAC1 genes were constructed by cloning the sequence of the coding region using the appropriate primers (**Table S2**) and inserting the fragment into the pcDNA3.1 (+) plasmid. The cells were transfected with the plasmids using Lipofectamine 2000 Transfection Reagent (Invitrogen) and then the medium was changed 6h after transfection.

### RNA extraction and quantitative real-time PCR

Total RNA was isolated using the TRIzol (Invitrogen) RNA extraction method, following the manufacturers’ instructions. qRT-PCR measurements were performed as described previously (17) with the appropriate primers listed in **Table S3**. Glyceraldehyde-3-phosphate dehydrogenase (GAPDH) was regarded as the internal reference, and the 2^−ΔΔCt^ method was applied to express the ratio of the target gene expression in the experimental group compared to the control group.

### Protein extraction and western blotting

Protein extraction and western blotting analysis were performed using previously standard procedures (17). The following antibodies were used for western blotting: anti-E-cadherin antibody(#20874-1-AP Proteintech, China), anti-N-cadherin antibody(#22018-1-AP Proteintech, China), anti-ZEB1 antibody(#66279-1-Ig Proteintech, China), anti-Vimentin antibody(10366-1-AP Proteintech, China), anti-SOX2 antibody(#11064-1-AP Proteintech, China), anti-GAPDH antibody(#10494-1-AP Proteintech, China), and anti-PD-L1 antibody(#66248-1-Ig Proteintech, China).

### Proliferation assay

Cell proliferation was detected using the Cell Counting Kit-8 (CCK-8, #K1018, APExBIO, USA) and the EdU cell proliferation assay kit (#C0071S, Beyotime Biotech, China), following the manufacturer’s instructions. The colony-formation assay was also performed to assess cell proliferation. 5000 cells were plated per well in triplicate in 6-well plates. The culture medium was changed every 3 days. Once visible clones were observed, each well was washed with PBS three times, fixed with methanol for 30 minutes at room temperature, and then stained with 0.05% crystal violet for 30 minutes. After washing, the colonies were counted and imaged.

### Migration and invasion assay

According to the published method (18), transwell migration (without Matrigel) and Matrigel (Matrigel, Corning, China) invasion assays were performed to evaluate cell migration and invasion abilities, respectively. Additionally, cell migration was measured using wound healing assays as previously described (18).

### Sphere formation assay

A sphere formation assay was performed to assess the stemness properties of LM3 and MHCC-97H cells. 1×10^5^ cells were seeded into the 6-well ultra-low attachment plates (Corning, China) in sphere formation medium (#CCM0012, Minneapolis, USA). The cells were incubated in a CO_2_ incubator for two weeks, and the number of spheres was counted under a stereomicroscope (Olympus).

### Statistical analysis

Statistical analysis was performed using R Studio (R version 4.2.3) and GraphPad Prism 9.0.2. For genes with multiple probes, the maximum expression was selected. Cox regression analysis was conducted using the “survival” package to assess the association between variables and prognosis, including hazard ratios and 95% confidence intervals (CI). Lasso analysis was employed as a variable selection method to refine the scope of variables, with the lambda value chosen for optimal regularization. All data were presented as the mean and standard error of the mean (mean ± SD, n = 3). GraphPad Prism was used to create bar graphs. A two-tailed Student’s t-test was used to compare the means between two groups, and an ANOVA test was used to assess significant differences among various experimental groups. The p-values in multiple comparisons were adjusted to control the false discovery rate (FDR) using the Benjamini-Hochberg method. The OS was evaluated using a Kaplan-Meier (K-M) curve, with statistical significance assessed using a log-rank test. The correlation between two variables conforming to a normal distribution was calculated employing the Pearson method. *P*< 0.05 was considered as statistically significant.

## Results

### Identification of prognostic-related genes

**Figure 1:**
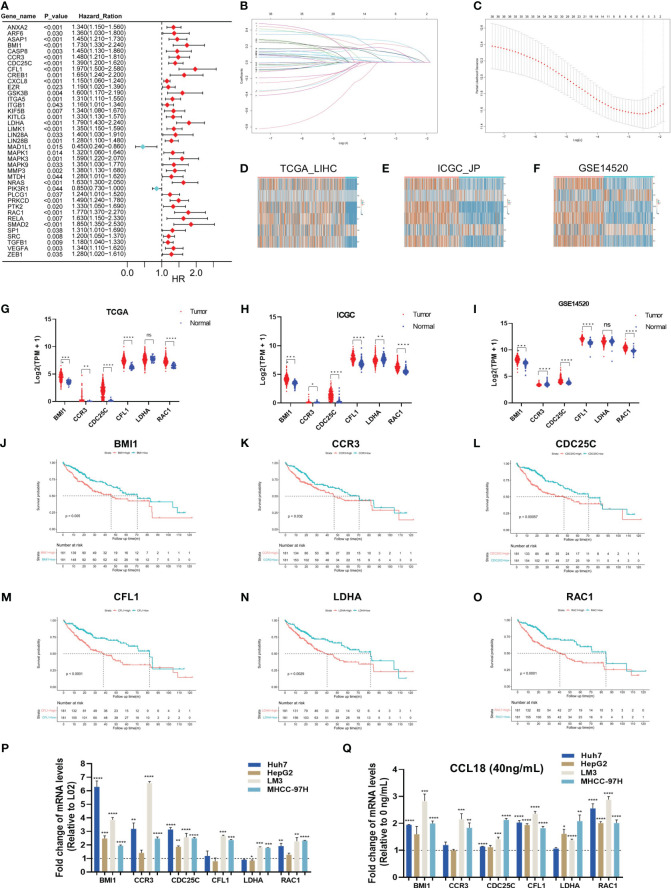
Six hub genes associated with prognosis in CCL18 signaling pathway in HCC. **(A)** Univariate Cox regression analysis for CCL18 signaling pathway-related genes in TCGA_LIHC training cohort. **(B**, **C)** The hub genes (n=6) were determined by the minimum lambda value of the LASSO regression analysis. **(D–I)** The heatmaps **(D–F)** and box plots **(G–I)** showed the transcription expression of six hub genes in TCGA_LIHC, ICGC_JP, and GSE14520 cohorts, consistently. **(J–O)** Survival analysis showed that all hub genes were associated with shorter survival. **(P)** RT-qPCR analysis of hub genes expression levels in L02, Huh7, HepG2, LM3 and MHCC-97H cells. **(Q)** RT-qPCR analysis of hub genes levels in Huh7, HepG2, LM3 and MHCC-97H cells after 48h of no stimulation or stimulation with 40 ng/mL CCL18. All experiments were performed with three experimental replicates, each measured with qPCR once. *P<0.05, **P<0.01, ***P<0.001, ****P<0.0001.

HCC raw datasets were obtained from the TCGA, ICGC, and GEO databases. Prior to analysis, these datasets were normalized using the log2(TPM + 1) transformation. 99 genes within CCL18 signaling pathway were downloaded from WikiPathways (**Table S1**). The expression patterns of these genes in the TCGA_LIHC cohort (n = 424) were illustrated using a heatmap (**Figure S1A**). Next, 39 genes exhibiting significant differences in hazard ration (HR) were identified from the TCGA_LIHC cohort by univariate Cox regression analysis (**Figure 1A**). Six hub genes, namely BMI1, CCR3, CDC25C, CFL1, LDHA, and RAC1, were identified using Lasso regression analysis (**Figures 1B, C**). Then, we compared the expression of these six hub genes in TCGA_LIHC, ICGC_JP (n = 445), and GSE14520 (n = 445) cohorts between normal and tumor samples (**Figures 1D–I**). The high expression of CCR3 and LDHA in tumor tissue is not prominent. This study shows that BMI1, CCR3, CDC25C, CFL1, and RAC1 consistently have higher expression levels, while LDHA only shows increased expression in ICGC_JP, not in TCGA_LIHC and GSE14520 cohorts. Additionally, the OS analysis revealed that patients with high expression of 6 hub genes exhibited a shorter survival time (**Figures 1J–O**) in the TCGA_LIHC cohort. Similarly, the Kaplan-Meier Plotter analyzed the correlation between the expression of six hub genes and survival, including OS, RFS, PFS, DSS (**Figures S1B–E**), and the results basically indicated that hub gene expression is associated with a poor prognosis in patients with HCC. On the other hand, the RT-qPCR assay was applied to detect the expression of hub genes in L02 normal hepatocytes and Huh7, HepG2, LM3, and MHCC-97H hepatoma cells (**Figure 1P**). Six hub genes were significantly up-regulated in LM3 and MHCC-97H cell lines, while only BMI1 and CDC25C were up-regulated in hepG2 cells, and CFL1 and LDHA did not show up-regulation in Huh7 cells. Additionally, the expression of hub genes was significantly upregulated upon stimulation with CCL18 in most HCC cells (**Figure 1Q**).

### Construction and verification of prognostic model

**Figure 2:**
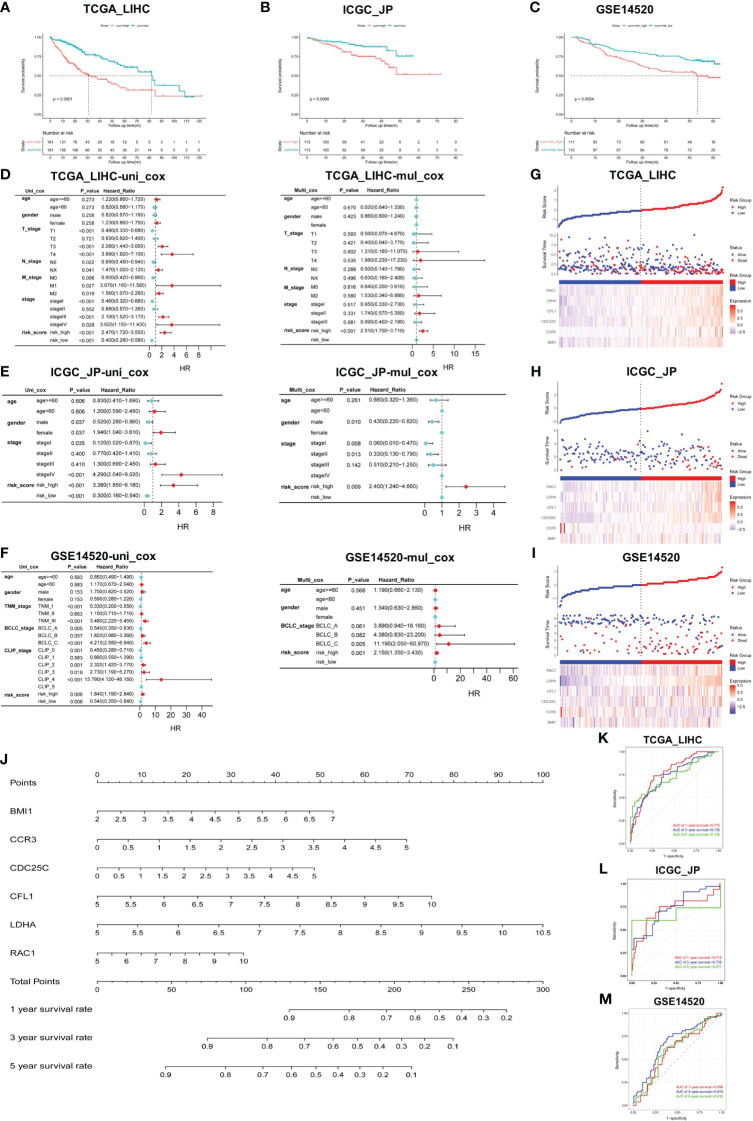
Construction and verification of prediction model. **(A–C)** Validation of risk score in TCGA_LIHC, ICGC_JP, and GSE14520 cohorts for OS. **(D–F)** Univariate and multivariate Cox regression analysis between risk score and other clinical characteristics in all cohorts. **(G–I)** Triplet graph showed the relationship between risk score, survival status, and gene expression. **(J)** The prognostic nomogram was built based on the 6 hub genes using TCGA_LIHC cohort. **(K–M)** The ROC curve for the prognostic performance of the nomogram in each cohort, including TCGA_LIHC **(K)**, ICGC_JP **(L)**, and GSE14520 **(M)**.

We calculated the risk score of individual HCC patients in all cohorts using the following formula: Risk score = [BMI1 expression × (0.1971976)] + [CCR3 expression × (0.2586062)] + [CDC25C expression × (0.181479)] + [CFL1 expression × (0.2796217)] + [LDHA expression × (0.3388622)] + [RAC1 expression × (0.1223471)]. Applying the median score as the best cut-off value, patients were divided into two groups: the high- and low-risk group. The K-M survival curve analysis revealed that the risk score served as a robust prognostic indicator for HCC patients. Notably, patients with higher risk scores exhibited significantly worse prognosis compared to those in the low-risk group (TCGA_LIHC *p*<0.001; ICGC_JP *p*<0.01; GSE14520 *p*<0.01) (**Figures 2A–C**).

To assess the potential of the risk score as an independent prognostic factor, we conducted univariate and multivariate Cox regression analyses to examine its association with other clinical characteristics, such as age, gender, and stage. We found that age and gender were not identified as independent predictors of prognosis in HCC patients, and the risk score served as an independent risk factor for OS in all cohorts (*p*<0.01) (**Figures 2D–F**). Risk score triptychs show the corresponding risk score, gene expression, and survival status (**Figures 2G-I**).

Additionally, a predictive nomogram was constructed using the TCGA_LIHC cohort, incorporating the expression levels of the six hub genes. This nomogram provided a quantitative assessment of the 1-, 3-, and 5-year survival rates for each HCC patient (**Figure 2J**), thereby offering potential clinical utility. The ROC curves showed the excellent predictive performance of the hub genes (**Figures 2K–M**), with area under the curve (AUC) values of 0.772 at 1-year, 0.735 at 3-year, and 0.725 at 5-year in the training cohort (**Figure 2K**). Moreover, the calibration curves were drawn to evaluate the consistency between the predictive survival possibility and the actual probability in TCGA_LIHC, ICGC_JP and GSE14520 cohorts (**Figures S2A–H**). These results highlight the remarkable precision and accuracy of the constructed nomogram.

### Exogenous CCL18 enhanced HCC cells’ proliferation, migration, invasion and stem cell-like phenotype

CCL18 exerts distinct effects in various cancer types, demonstrating its ability to enhance the proliferation of specific malignancies, such as ovarian cancer and osteosarcoma. Furthermore, CCL18 acts as an inducer, promoting tumor metastasis^13^. However, the effects of CCL18 on HCC cells have not been deeply studied. Hence, our study aimed to investigate the effects of exogenous CCL18 on HCC cells.

**Figure 3:**
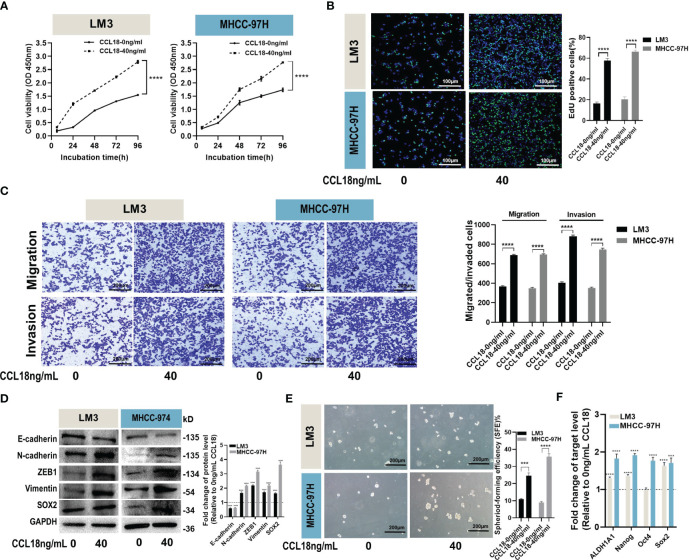
CCL18 promotes proliferation, migration, invasion and stemness properties of HCC cells *in vitro*. **(A, B)** Proliferation of LM3 and MHCC-97H cells after 48h of no stimulation or stimulation with 40 ng/mL CCL18 was examined by CCK-8 assays **(A)** and EdU assays (**(B)**, Scale bar:100 μm). **(C)** Migration and invasion of LM3 and MHCC-97H after 48h of no stimulation or stimulation with 40 ng/mL CCL18 were detected by Transwell assays. Scale bar:200 μm. **(D)** The protein levels of EMT and stemness related gene in LM3 and MHCC-97H cells after 48h of no stimulation or stimulation with 40 ng/mL CCL18. **(E)** Sphere-formation abilities of LM3 and MHCC-97H cells were assessed after 48h of no stimulation or stimulation with 40 ng/mL CCL18. Scale bar:200 μm. **(F)** The expression of stemness-related genes in LM3 and MHCC-97H cells after 48h of no stimulation or stimulation with 40 ng/mL CCL18. All data are shown as the mean ± SD. *P<0.05, **P<0.01, ***P<0.001, and ****P<0.0001 by two-tailed Student’s *t*-test.

To investigate the biological functions of CCL18 in LM3 and MHCC-97H cells, we stimulated the cells with or without 40ng/mL CCL18 for 48h observed its impact on cell proliferation, migration, invasion and stemness properties. CCK-8 and EdU assays revealed that CCL18 promoted the proliferation abilities of LM3 and MHCC-97H cells (**Figures 3A, B**). Transwell migration and invasion assays showed that CCL18 significantly promoted the migration and invasion abilities of LM3 and MHCC-97H cells (**Figure 3C**). Alterations in the expression of Epithelial-Mesenchymal Transition (EMT)-associated proteins were detected by western blotting, indicating that CCL18 facilitated the EMT process (**Figure 3D**). Cell stemness was determined by the sphere formation assay (**Figure 3E**) and the detection of stemness gene expressions (**Figure 3F**). The results suggest that CCL18 promotes stemness of LM3 and MHCC-97H cells. Taken together, these data collectively indicate that CCL18 may play a promoting role in HCC cell proliferation, migration, invasion, and stemness *in vitro*.

### PPI construction of high-risk tumor samples and functional enrichment analysis

**Figure 4:**
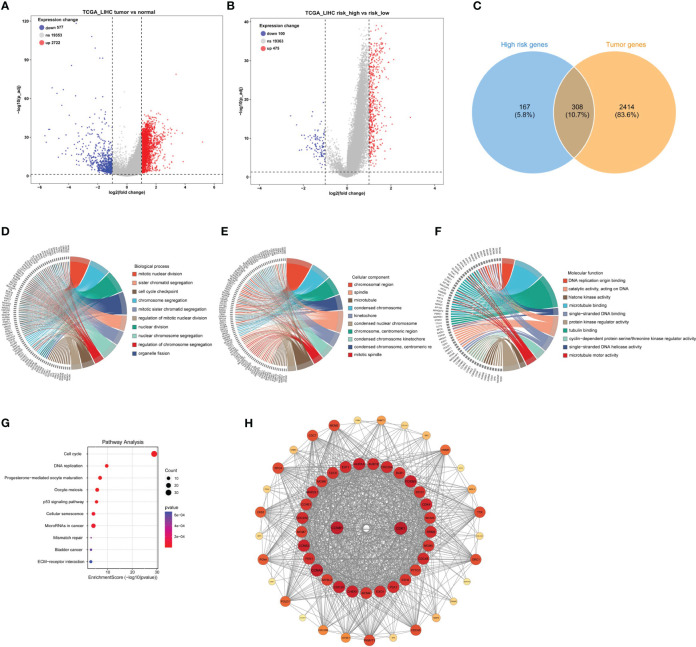
Identification of DEGs in tumors with high-risk samples and PPI functional enrichment analysis. **(A)** The volcano plot showing the DEGs in the high- and low-risk scores in TCGA_LIHC cohort. **(B)** The volcano plot revealed the DEGs in tumor and normal samples in TCGA_LIHC cohort. **(C)** The Venn plot displayed the intersection of high-risk score samples and tumor samples. **(D–G)** The chord diagrams showed the results of GO enrichment analysis, including biological process **(D)**, molecular function **(E)**, and cellular component **(F)**, and KEGG result **(G)**. **(H)** The key module analyzed by MODE included 104 genes out of the 308 genes shown by the Cytoscape software.

To investigate the genomic composition of the high-risk group in tumors, we reanalyzed the TCGA_LIHC cohort. Applying the thresholds of p< 0.05 and log2FoldChange > 1, we identified a total of 2722 DEGs in HCC tumor samples (**Figure 4A**) and 475 DEGs in the high-risk group (**Figure 4B**). By intersecting these two gene sets, we identified a total of 308 genes (**Figure 4C**).

Next, to better understand the function and specific mechanism of these genes, we utilized the “clusterProfiler” R package to conduct GO and KEGG enrichment analysis. The analysis of biological processes indicated a significant enrichment of genes specifically expressed in the high-risk group of tumors in processes such as “mitotic nuclear division” and “sister chromatid segregation” (**Figure 4D**). Furthermore, the analysis of cellular components highlighted the enrichment of these genes in cellular locations such as the “chromosomal region” and “spindle” (**Figure 4E**). Moreover, the molecular function analysis demonstrated a significant enrichment of these genes in functions such as “DNA replication origin binding” and “catalytic activity, acting on DNA” (**Figure 4F**). Additionally, the KEGG pathway analysis indicated a significant enrichment of these genes in pathways such as “cell cycle” and “DNA replication” (**Figure 4G**). Furthermore, the interaction network of the 308 proteins was analyzed using the STRING database. Subsequently, the MCODE plugin identified a highly significant cluster consisting of 104 proteins (**Figure 4H**). This observation suggests that these 104 proteins play a crucial role as the main regulatory agents within the high-risk group of HCC.

### Evaluation of immune cell infiltration

**Figure 5:**
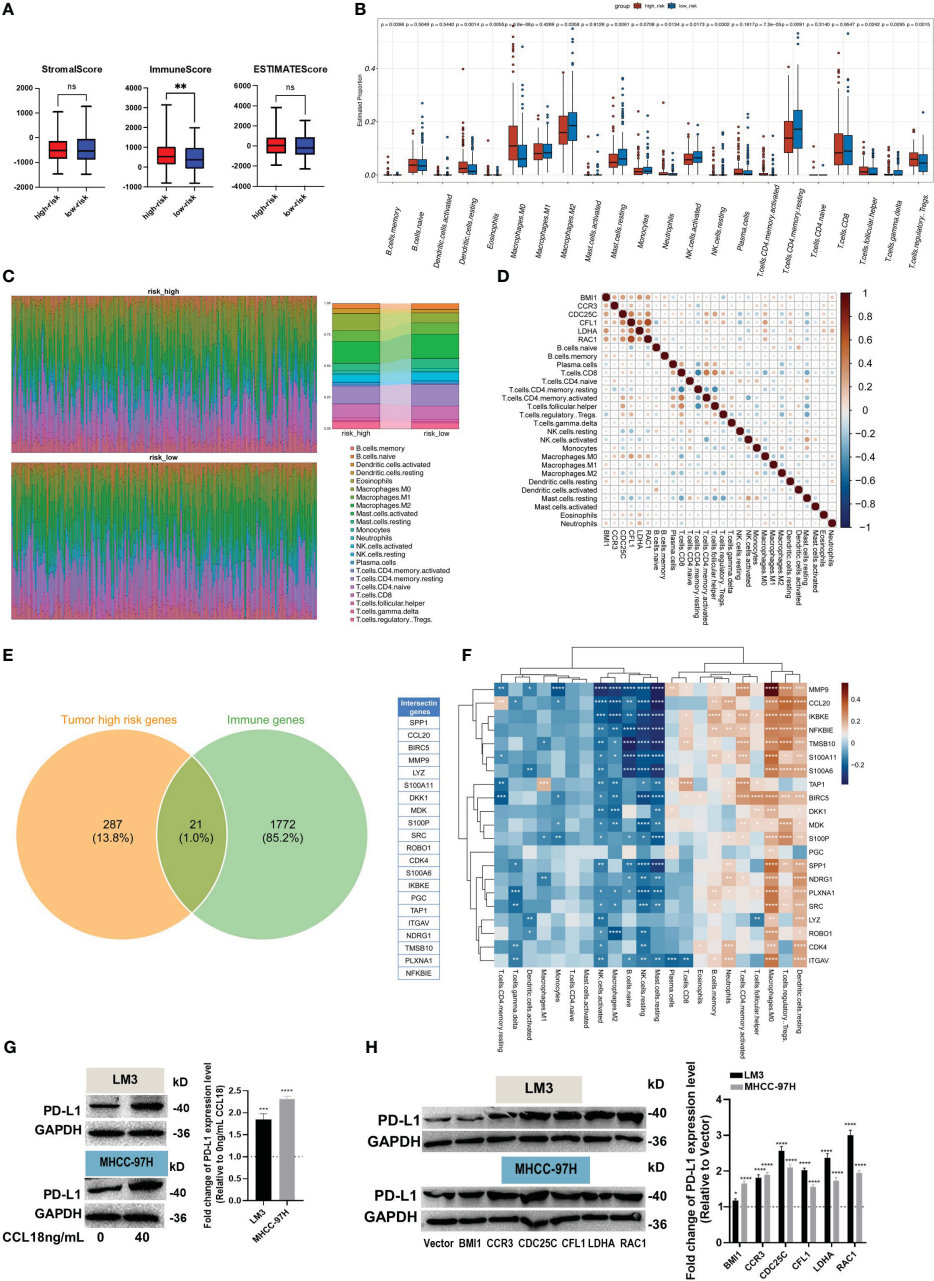
Evaluation of immune infiltration and immune escape. **(A)** The Estimate R package was used to calculate stromal, immune, and ESTIMATE scores. **(B, C)** The box plot **(B)** showed the different levels of 22 immune cell subtypes, and the relative proportion of immune cells is shown by the accumulation diagram **(C)**, both in the CIBERSORT package. **(D)** The correlation between hub genes and immune cells. **(E)** The Venn plot displays the 21 immune-related genes in high-risk samples. **(F)** The correlation heat map shows the correlation of 21 immune-related genes with 22 immune cells. **(G, H)** The protein levels of PD-L1 in LM3 and MHCC-97H cells were measured after stimulation without or with 40 ng/mL CCL18 for 48h **(G)**, or transfection with BMI1, CCR3, CDC25C, CFL1, LDHA and RAC1 overexpression plasmids for 48h **(H)**. *P<0.05, **P<0.01, ***P<0.001, ****P<0.0001.

The direct chemotactic effect of CCL18 on Treg cells and its role in modulating the immunosuppressive tumor microenvironment are well-established (19). Using the ESTIMATE R package, we performed calculations of the relevant indicators for both the high- and low-risk groups. Our results revealed no significant differences in the Stromal Score and ESTIMATE Score values between the high- and low-risk groups. However, a notable disparity was observed in the Immune Score values, indicating a significantly higher level of immune cell infiltration in the tumor samples from the high-risk group (**Figure 5A**). This finding provides evidence that the high-risk group of tumor samples exhibits a greater extent of immune cell infiltration compared to the low-risk group.

Additionally, the CIBERSORT R package was employed to investigate the composition of immune cells and explore the correlation between hub genes and immune cells. The box plots illustrate the estimated proportions of 22 immune cell types in both the high- and low-risk groups (**Figure 5B**). The findings suggest that immune suppression may indeed occur in the high-risk group of HCC. This conclusion is supported by the higher relative abundance of Memory B cells, resting dendritic cells, eosinophils, M0 macrophages, neutrophils, T cells CD4 memory resting, T cells follicular helper, and Tregs observed in this group. Conversely, the low-risk group exhibits a higher relative abundance of M2 macrophages, resting mast cells, activated/resting NK cells, resting T cells CD4 memory, and T cells gamma delta. The scatter plot shows the proportions of the 22 immune cells at the individual patient level (**Figure 5C**). Additionally, the Pearson correlation coefficient was calculated to assess the correlation between hub genes and immune cell types (**Figure 5D**). The Venn diagram illustrates the high expression of 21 immune-related genes in the high-risk group of tumor samples (**Figure 5E**). We computed the correlation between these genes and immune cell types, and the results were similar to previous findings (**Figure 5F**).

Eventually, up-regulation in PD-L1 protein expression was observed in HCC cells after stimulation of CCL18 (**Figure 5G**) or transfection with CCR3, CDC25C, CFL1, LDHA or RAC1 plasmid (**Figure 5H**). Together, these results imply that CCL18 signaling pathway is associated with immune cell infiltration and immune escape in the HCC microenvironment.

### Functional analysis of hub genes on proliferation, migration, invasion and stemness of HCC cells

**Figure 6:**
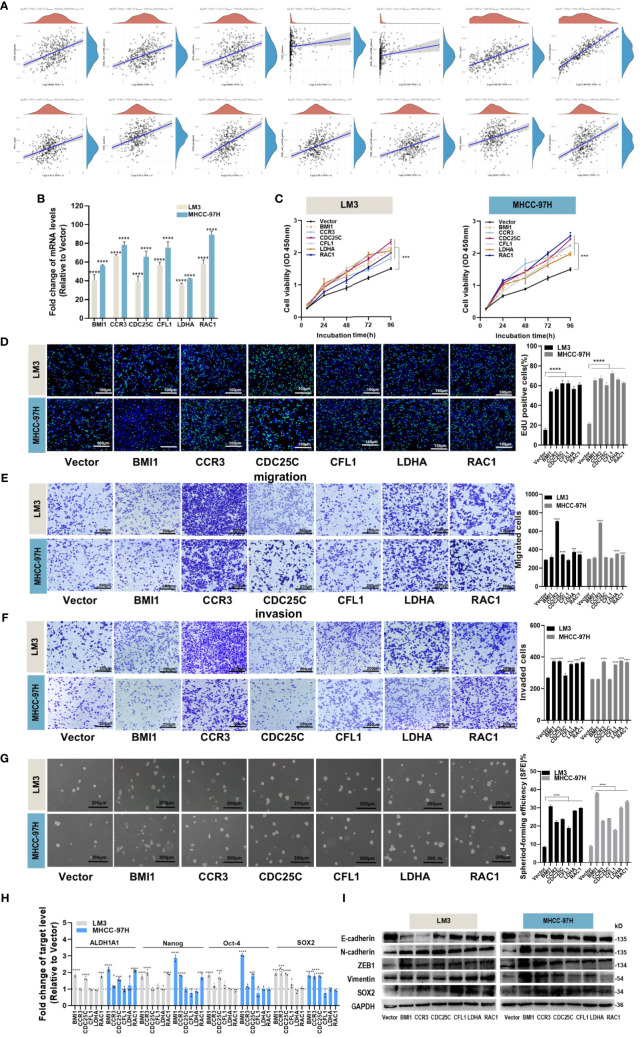
Effects of hub genes on proliferation, migration, invasion and stemness properties in HCC cells. **(A)** The correlations between six hub genes and pathway score were analyzed using Spearman. The abscissa represents the distribution of gene expression, and the ordinate represents the distribution of pathway score. The density curve on the right represents the trend in the distribution of pathway immune score, while the upper density curve represents the trend in the distribution of gene expression. The value on the top represents the correlation p value, correlation coefficient and correlation calculation method. **(B)** The overexpression efficiency of BMI1, CCR3, CDC25C, CFL1, LDHA and RAC1 overexpression plasmid was assessed by RT-qPCR. **(C, D)** Proliferation of LM3 and MHCC-97H cells after transfection with BMI1, CCR3, CDC25C, CFL1, LDHA and RAC1 overexpression plasmid for 48h was examined by CCK-8 assays **(C)** and EdU assays (**(D)**, Scale bar:100 μm). **(E, F)** Migration **(E)** and invasion **(F)** of LM3 and MHCC-97H cells after transfection with BMI1, CCR3, CDC25C, CFL1, LDHA and RAC1 overexpression plasmid for 48h were detected by Transwell assays. Scale bar:200 μm. **(G)** Sphere-formation abilities of LM3 and MHCC-97H cells were observed after transfection with BMI1, CCR3, CDC25C, CFL1, LDHA, and RAC1 overexpression plasmids for 48h. Scale bar: 200 μm. **(H)** The expression of stemness-related genes in LM3 and MHCC-97H cells was analyzed after transfection with BMI1, CCR3, CDC25C, CFL1, LDHA, and RAC1 overexpression plasmids for 48h. **(I)** The protein levels of EMT-related genes in LM3 and MHCC-97H cells were measured after transfection with BMI1, CCR3, CDC25C, CFL1, LDHA, and RAC1 overexpression plasmids for 48h. All data are shown as the mean ± SD. *P<0.05, **P<0.01, ***P<0.001 and ****P<0.0001 by two-tailed Student’s t-test.

We performed an analysis to examine the correlation between six hub genes and various signaling pathways. By applying a significance threshold of p< 0.05 and |cor| > 0.3, we successfully identified specific hub genes associated with different pathways. Within the DNA replication pathway, we found BMI1, CDC25C, and CFL1 to be the hub genes of interest. Similarly, the G2M pathway revealed the presence of BMI1, CDC25C, CFL1, and RAC1 as significant hub genes. Moving to the PI3K pathway, our analysis highlighted BMI1, CCR3, CFL1, and LDHA as the hub genes involved. Lastly, within the EMT pathway, the hub genes CCR3, CFL1, and RAC1 were found to play crucial roles (**Figure 6A**).

In order to elucidate the biological functions of the hub genes, various analyses including proliferation, migration, invasion, and stemness were conducted on HCC cells. Overexpression plasmids of BMI1, CCR3, CDC25C, CFL1, LDHA, and RAC1 were constructed and subsequently transfected into LM3 and MHCC-97H cells. The transfection efficiency was confirmed through qPCR analysis (**Figure 6B**). The CCK-8 and EdU assays demonstrated that all the aforementioned genes played a role in promoting HCC cell proliferation (**Figures 6C, D**). Transwell migration and invasion assays suggested that overexpression of CCR3, LDHA, and RAC1 enhanced the migration and invasion abilities of both LM3 and MHCC-97H cells (**Figures 6E, F**). The results of the sphere formation assay revealed that all of the above genes supported the maintenance of stemness properties in HCC cells (**Figure 6G**), and the detection of stemness marker genes suggested that BMI1, CCR3, CDC25C, and RAC1 maintained the stemness of LM3 and MHCC-97H cells by upregulating stemness transcription factors (**Figure 6H**). Western blot experiments showed that overexpression of BMI1, CCR3, CDC25C, CFL1, LDHA, and RAC1 could induce EMT (**Figure 6I**).

Together, these results imply that different hub genes are involved in different processes that contribute to HCC progression.

### Identification of candidate agents in high-risk score patients

**Figure 7:**
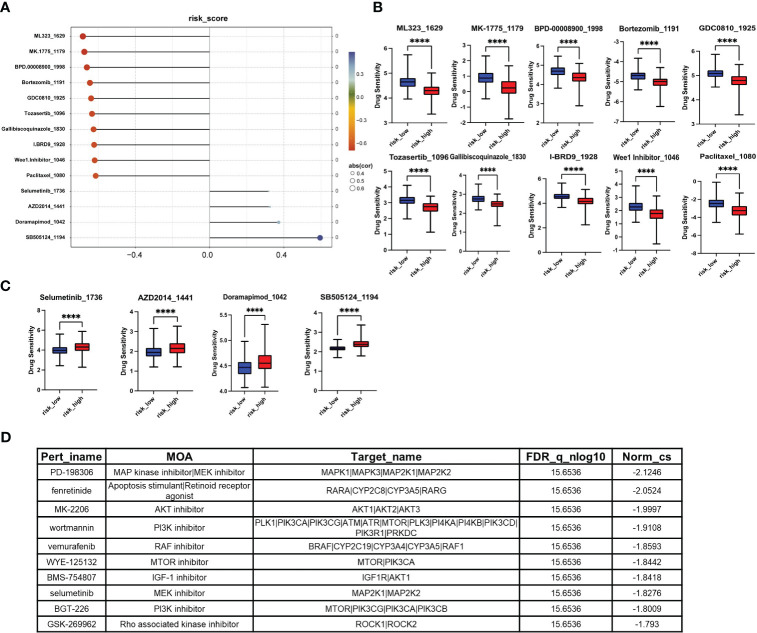
Screening of small molecule compounds with potential therapeutic effects in high-risk group. **(A)** The lollipop chart shows the results of OncoPredict R package according to p<0.05, cor>0.3 or cor<-0.6. **(B)** Potential drugs for the treatment of high-risk HCC patients. **(C)** Potentially inappropriate drugs for the treatment of high-risk HCC patients. **(D)** The top 10 compounds with the highest negative scores according to CAMP. *P<0.05, **P<0.01, ***P<0.001, ****P<0.0001.

To provide better clinical recommendations, we calculated the sensitivity score of common drugs in TCGA_LIHC patients using the “OncoPredict” R package. We then calculated the Pearson correlation between the drug sensitivity score and risk score. Based on a significance level of p<0.05 and a correlation coefficient of cor>0.3 or cor<-0.6, we selected 14 anti-tumor drugs for horizontal lollipop mapping (**Figures 7A–C**). Meanwhile, we utilized the CAMP database to further predict potential small molecule compounds for the treatment of high-risk group patients. Based on the lowest scores, we identified the top 10 ranking compounds as PD-198306, fenretinide, MK-2206, wortmannin, vemurafenib, WYE-125132, BMS-754807, selumetinib, BGT-226 and GSK-269962 (**Figure 7D**). However, it is important to note that further experimental validation and in-depth studies are required to confirm the therapeutic potential and efficacy of these compounds.

## Discussion

HCC is a highly malignant cancer that requires immediate investigation of new therapeutic strategies. Traditional first-line treatment drugs, such as Sorafenib and Lenvatinib, have only shown slight improvements in OS, with an extension of approximately 2.8 and 4.4 months (3). Over the past five years, significant advancements have been made in the field of immunotherapy, specifically in ICIs. However, in advanced HCC patients, monotherapy with ICIs has only demonstrated objective response rates of 15-20%, without any significant improvement in OS. Furthermore, specific biomarkers for this subgroup of patients have yet to be identified (20). The tumor microenvironment of HCC is characterized by a significant presence of non-tumor stromal cells, including cancer-associated fibroblasts, endothelial cells, TAMs, B cells, and T cells. These cells play crucial roles in the progression of cancer (7). Among these cells, our particular focus lies on the role of macrophages.

CCL18, also known as macrophage inflammatory protein 4 (MIP-4), pulmonary and activation-regulated chemokine (PARC), dendritic cell chemokine 1 (DC-CK1), and alternative macrophage activation-associated CC chemokine 1 (AMAC-1), belongs to the family of CC chemokines and acts as a chemoattractant. CCL18 is located on chromosome 17 in the human genome and shares the highest amino acid identity (65%) with CCL3. It encodes a protein consisting of 89 amino acids, with the mature active form comprising 69 amino acids without a terminal alanine at the C-terminus (13, 21). In recent years, more and more studies have revealed the existence of multiple subtypes within M1 and M2 macrophages, including further subdivisions of M2 macrophages into M2a, M2b, M2c, and M2d subgroups (14). However, the specific M2 macrophage subtype responsible for secreting CCL18 remains to be conclusively determined. The progression of cancer necessitates evasion of immune surveillance, and CCL18, which is secreted by M2 macrophages, serves as a hallmark of macrophage activation. It acts as a chemotactic factor that promotes immune suppression and immune escape, thereby facilitating tumor development (13). Studies have demonstrated the crucial role of CCL18 in the progression of fibrotic immune diseases (22) and tumors. CCL18 has been shown to promote immunosuppressive states and progression in esophageal squamous cell carcinoma (23), multiple myeloma (24), osteosarcoma (25), ovarian cancer (26), and renal cell carcinoma (27). In a word, the CCL18 signaling pathway has demonstrated its prognostic significance in patients with tumors.

Several gene prognostic models related to signaling pathways have been reported in HCC, including STING pathway genes (28), hypoxia-related and immune-associated genes (29), and chromatin organization-related genes (30). However, the biological functions and prognostic impact of CCL18 signaling pathway genes in HCC remain largely unknown. In this study, we acquired CCL18 signaling pathway-associated genes from the Wikipathway website and obtained RNA-seq sequencing data of HCC from publicly available databases such as TCGA, ICGC, and GEO. By employing a diverse range of well-established bioinformatics methodologies, we successfully identified six key genes (BMI1, CCR3, CDC25C, CFL1, LDHA, RAC1) that have a substantial impact on prognosis. Subsequently, we validated the independent prognostic value of the expression of these genes in predicting the prognosis of HCC patients through multivariate Cox regression analysis. Furthermore, we utilized these genes to construct a nomogram that enables the prediction of patients’ OS rates at 1-, 3-, and 5- years. In summary, these findings provide robust evidence supporting the prognostic evaluation and personalized treatment of HCC, thereby contributing to the enhancement of patients’ survival rates and treatment efficacy. Additionally, we analyzed the core protein network of highly expressed proteins in the high-risk group and compared the differences in immune cell infiltration between the high- and low-risk groups. The results revealed a higher proportion of immune-inhibitory cells in the high-risk group, suggesting that the overexpression of these hub genes indeed induces immune suppression in tumors. In the experimental section, we investigated the effects of exogenous CCL18 on the biological functions of LM3 and MHCC-97H cells. We observed that exogenous CCL18 promoted cell proliferation, migration, invasion, and stemness. Additionally, by overexpressing hub genes in the cell lines, we identified that BMI1, CCR3, CDC25C, CFL1, LDHA, and RAC1 participated in promoting different functions of HCC cells. Furthermore, we discovered that both exogenous CCL18 and overexpression of CCR3, CDC25C, CFL1, LDHA, and RAC1 could induce the expression of PDL1 in the HCC cell lines, which is consistent with the occurrence of immune suppression in HCC. However, the molecular processes and regulatory mechanisms underlying these findings require further investigation in future studies.

BMI1, a polycomb-group protein, is involved in the regulation of embryonic development and DNA damage repair. It is also an oncogene, with dysregulated expression frequently associated with various cancers. H. Wang et al. discovered that exogenous CCL18 can promote the up-regulation of OCT4 and BMI1 mRNA and protein expression (31). Moreover, up-regulation of BMI1 expression in HCC has been linked to its role in blocking the INK4a/ARF locus, NF-κB signaling pathway, and TGFβ2/SMAD signaling axis, while simultaneously activating the Wnt/β-catenin signaling axis, thereby promoting the development of HCC (32). Inhibition of BMI1 has been shown to enhance immune checkpoint blockade in CCA cells (33). Additionally, BMI1 has been implicated in promoting breast cancer (34) and endometrial cancer (35).

The role of CCR3 in tumor cells is relatively limited, as it is primarily highly expressed in inflammatory cells such as mast cells, eosinophils, basophils, and Th2 cells. It plays a significant role in inflammatory responses (36). CCL18 acts as a neutral CCR3 antagonist. CCR3 exerts its functions through various ligands. In breast cancer, it promotes cancer progression through the CCL5-CCR3 axis (37). Compared to primary prostate tumors, CCR3 exhibits high expression in bone and visceral metastases, potentially exerting its effects via the CCR3/CCL7 axis (38). In renal cell carcinoma, CCR3 facilitates tumor proliferation and metastasis through the CCL11/CCR3 axis (39).

CDC25C is one of the three isoforms of the CDC25 phosphatase family, and it plays a crucial role in regulating the G2/M transition and mediating DNA damage repair during cell division. Extensive research has demonstrated that abnormal expression of CDC25C is associated with the progression of various types of cancer (40). CFL1, a 166-amino acid phosphoprotein, is one of the five components representing actin-binding proteins. It regulates the polymerization and depolymerization of F-actin and G-actin (41). Similarly, CFL1 also contributes to the proliferation, invasion, and metastasis of malignant tumors (42–44). The lactate dehydrogenase isoenzymes (LDH) are tetramers composed of LDHA and LDHB, and their aberrant expression is often associated with cellular metabolism and tumor progression. Chen et al. discovered that the expression of LDHA was upregulated and LDHB was downregulated in prostate cancer cells by exogenous CCL18 at both mRNA and protein levels (45). In addition, LDHA can also serve as a biomarker for various malignant tumors (46–48). RAC1, a small GTP-binding protein, belongs to the Rac subfamily of the Rho GTPase family, and it is involved in various biological functions, including regulating cell migration, signal transduction, and promoting cell polarization. Lihong Shi et al. discovered that elevated levels of CCL18 promote lymph node metastasis and distant metastasis in NSCLC patients. They demonstrated that CCL18 activates RAC1 to regulate cellular migration and invasion, ultimately leading to cytoskeletal remodeling *in vitro* (49). RAC1 has been extensively discussed for its role in promoting proliferation, participating in angiogenesis, facilitating tumor migration and invasion, as well as its involvement in stemness in tumor cells (50).

Immunotherapy remains a promising trend for HCC patients in the future. In our study, we found that exogenous CCL18, as well as CCR3, CDC25C, CFL1, LDHA or RAC1 plasmid could promote the production of PD-L1 protein in LM3 and MHCC-97H cells. This suggests that immune suppression may occur as a result. Furthermore, the high-risk group derived from the six hub genes also exhibits a positive correlation with immune-suppressive cells such as Treg cells.

Our study provides the first systematic elucidation of the six hub genes in the CCL18 signaling pathway that impact the prognosis of HCC patients. Additionally, we constructed a protein-protein interaction network of key proteins in the high-risk group and analyzed the immune cell infiltration in the high-risk group. These findings contribute to our understanding of immune evasion genes in HCC.

Our study has several limitations. Firstly, in terms of variable selection, it would be advantageous to explore a range of machine learning methods, such as random forest and support vector machines, to enhance the accuracy of our analysis. Secondly, among the identified hub genes, CDC25C, CFL1, and LDHA are primarily associated with abnormal physiological activities, such as cytoskeletal dynamics, cellular respiration, and cell replication and proliferation, rather than directly interacting with the CCL18 chemokine. Lastly, in our cellular experiments, we have primarily focused on functional experiments related to gene overexpression. To further deepen our understanding, future investigations should include knockdown and rescue experiments.

## Conclusion

In summary, our study conducted a comprehensive analysis of the prognostic impact of six hub genes within the CCL18 signaling pathway in HCC patients. Our investigation demonstrated that exogenous CCL18 enhances key oncogenic processes in HCC cell lines LM3 and MHCC-97H, including proliferation, migration, invasion, and up-regulation of the immune-suppressive marker PD-L1 protein. We also investigated the functions of six key genes, revealing their potential involvement in liver cancer development. We further identified 21 immune-related genes that exhibit strong correlations with immune suppressive cells. Collectively, these findings significantly contribute to our understanding of immune evasion within the tumor microenvironment and the underlying oncogenic processes in HCC patients.

## Data Availability

The original contributions presented in the study are included in the article/**Supplementary Material**. Further inquiries can be directed to the corresponding authors.
