# Correction: Identification of hub genes within the CCL18 signaling pathway in hepatocellular carcinoma through bioinformatics analysis

**DOI:** 10.3389/fonc.2025.1649612

**Published:** 2025-07-18

**Authors:** Jinlei Mao, Yuhang Tao, Keke Wang, Hanru Sun, Manqi Zhang, Liang Jin, Yi Pan

**Affiliations:** State Key Laboratory of Natural Medicines, Jiangsu Key Laboratory of Druggability of Biopharmaceuticals, School of Life Science and Technology, China Pharmaceutical University, Nanjing, Jiangsu, China

**Keywords:** hepatocellular carcinoma, CCL18, tumor microenvironment, prognostic, diagnosis

In the published article, there were errors in **Figure 6**. Due to carelessness during the preparation of the **Figures 6E** and **F**, the images labeled “CCR3” in the row for “Migration, LM3, MHCC-97H”, images labeled “LDHA” in the row for “Migration, LM3, MHCC-97H”, images labeled “CCR3”,”LDHA” in the row for “Invasion, LM3”, images labeled “CCR3”,”LDHA” in the row for “Invasion, MHCC-97H” and images labeled”Vector”,”CDC25C” in the row for “Invasion, MHCC-97H” show overlapping fields of view. We replaced images with the correct ones. The corrected **Figure 6** and its caption appear below.

**Figure 6:**
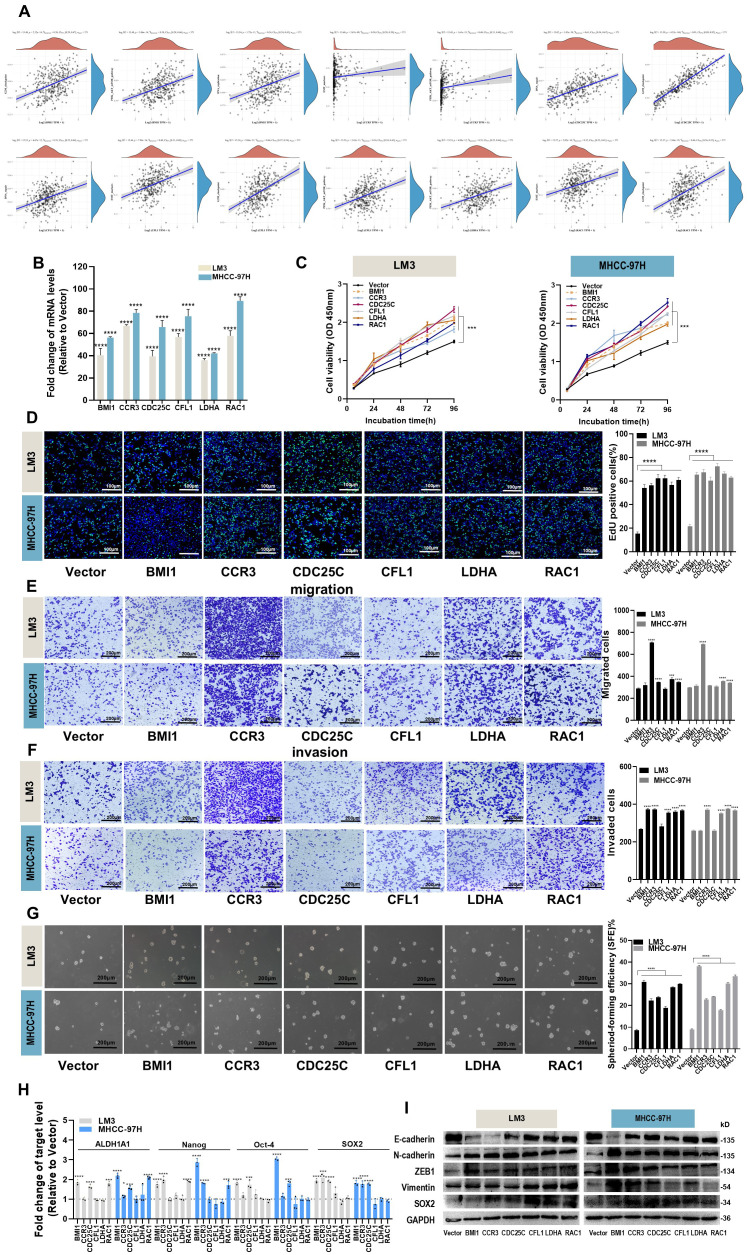
Effects of hub genes on proliferation, migration, invasion and stemness properties in HCC cells. **(A)** The correlations between six hub genes and pathway score were analyzed using Spearman. The abscissa represents the distribution of gene expression, and the ordinate represents the distribution of pathway score. The density curve on the right represents the trend in the distribution of pathway immune score, while the upper density curve represents the trend in the distribution of gene expression. The value on the top represents the correlation p value, correlation coefficient and correlation calculation method. **(B)** The overexpression efficiency of BMI1, CCR3, CDC25C, CFL1, LDHA and RAC1 overexpression plasmid was assessed by RT-qPCR. **(C, D)** Proliferation of LM3 and MHCC-97H cells after transfection with BMI1, CCR3, CDC25C, CFL1, LDHA and RAC1 overexpression plasmid for 48h was examined by CCK-8 assays **(C)** and EdU assays (**(D),** Scale bar:100 μm). **(E, F)** Migration **(E)** and invasion **(F)** of LM3 and MHCC-97H cells after transfection with BMI1, CCR3, CDC25C, CFL1, LDHA and RAC1 overexpression plasmid for 48h were detected by Transwell assays. Scale bar:200 μm. **(G)** Sphere-formation abilities of LM3 and MHCC-97H cells were observed after transfection with BMI1, CCR3, CDC25C, CFL1, LDHA, and RAC1 overexpression plasmids for 48h. Scale bar: 200 μm. **(H)** The expression of stemness-related genes in LM3 and MHCC-97H cells was analyzed after transfection with BMI1, CCR3, CDC25C, CFL1, LDHA, and RAC1 overexpression plasmids for 48h. **(I)** The protein levels of EMT-related genes in LM3 and MHCC-97H cells were measured after transfection with BMI1, CCR3, CDC25C, CFL1, LDHA, and RAC1 overexpression plasmids for 48h. All data are shown as the mean ± SD. *P<0.05, **P<0.01, ***P<0.001 and ****P<0.0001 by two-tailed Student’s t-test.

The original version of this article has been updated.

