# Supplementary material for: Identification of hub genes within the CCL18 signaling pathway in hepatocellular carcinoma through bioinformatics analysis
Source: Front Oncol. 2024 Mar 6;14:1371990. doi: 10.3389/fonc.2024.1371990 (PMC10952098; doi:10.3389/fonc.2024.1371990)
Supplement: Supplementary file 1 [file DataSheet1.docx]

**
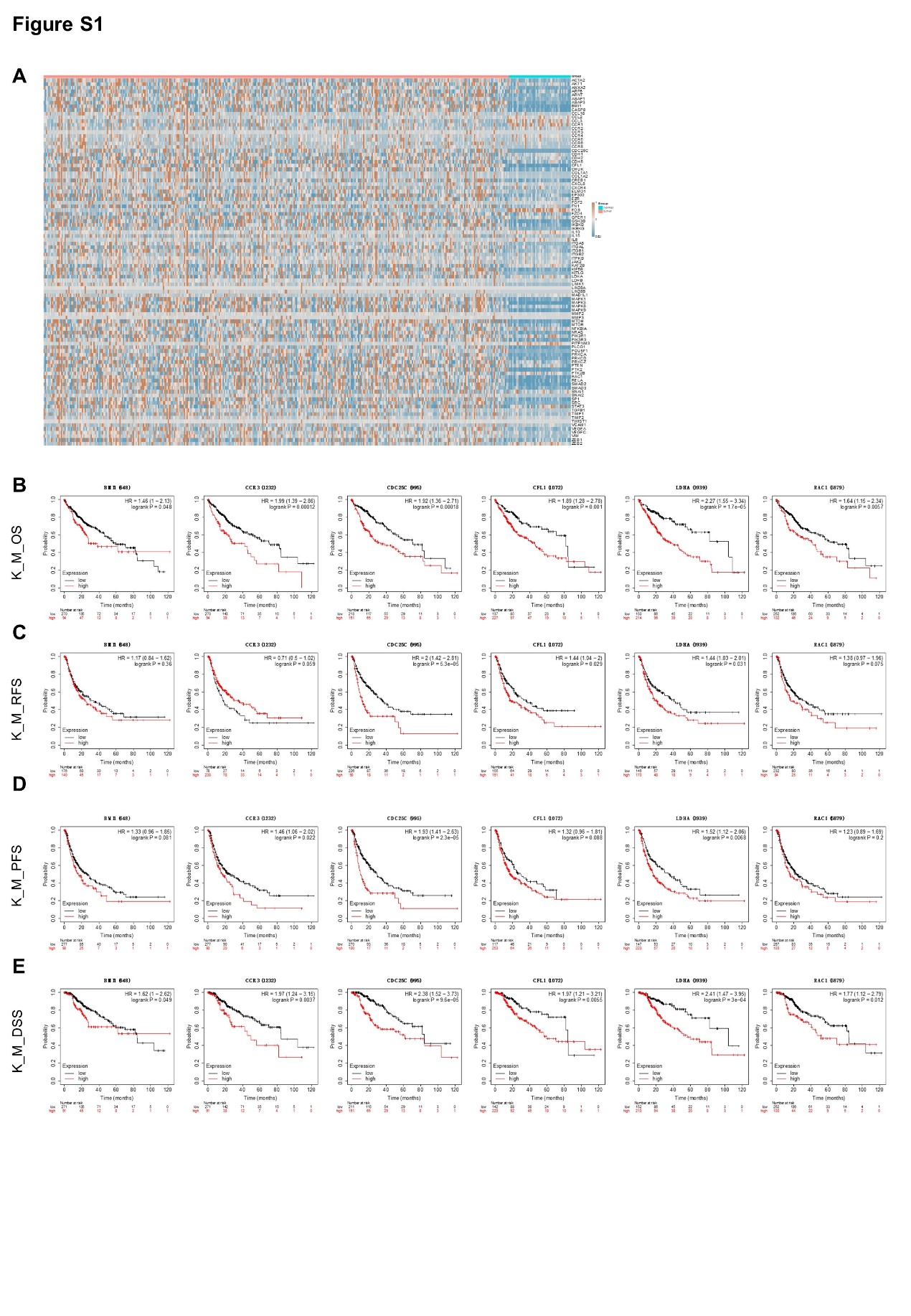
Supplementary Figure S1 Analysis of CCL18 pathway gene expression and hub genes survival rate.** **(A)** The heat map showed the expression of 99 genes of CCL18 signaling pathway in tumor tissue and normal tissue in TCGA liver cancer patients. **(B-E)** Kaplan-Meier Plotter website was used to analyze the Overall Survival (B), recurrence free survival (C), Progression Free Survival (D) and Disease-specific survival (E) of 6 hub genes.

**
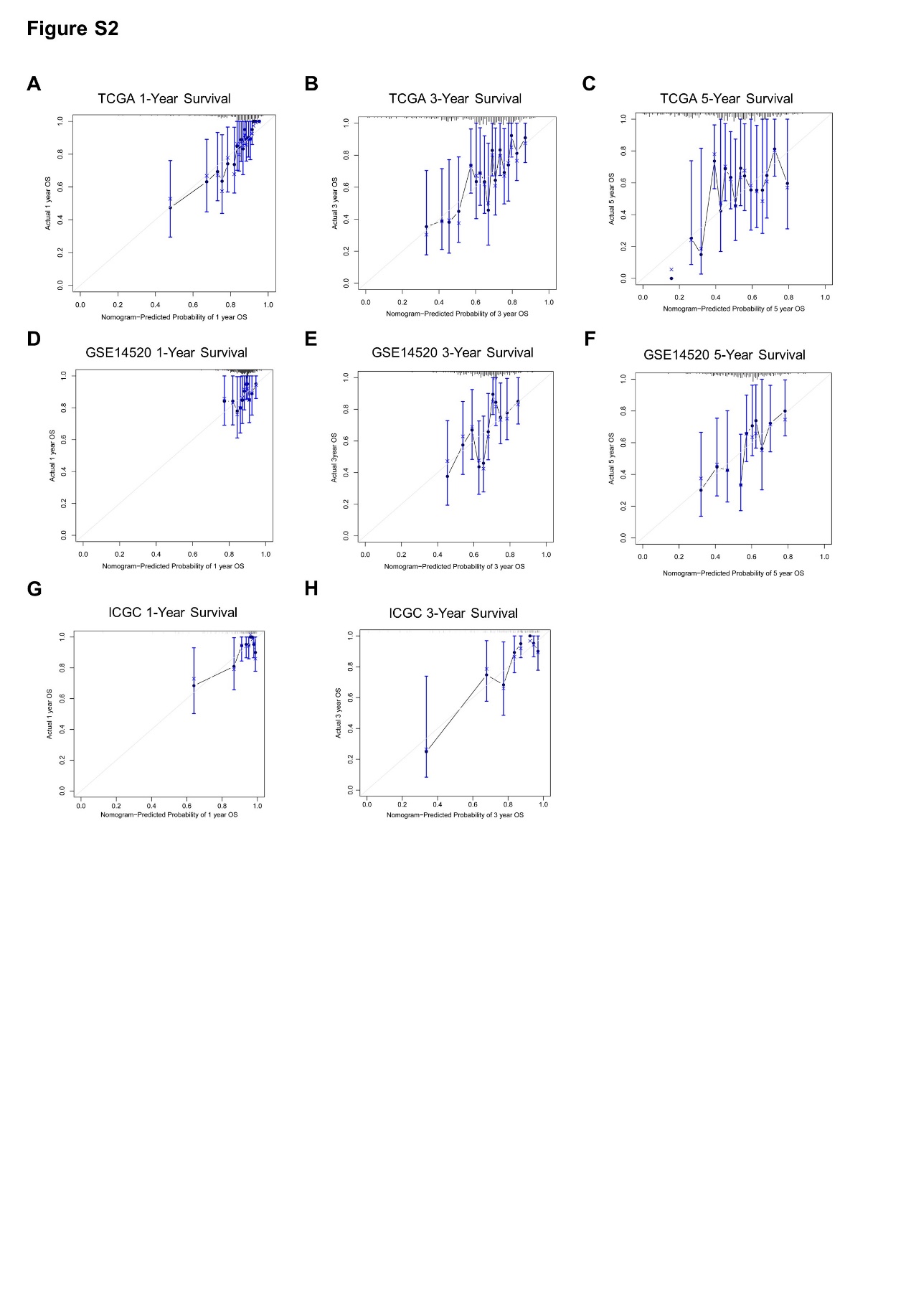
Supplementary Figure S2 Calibration Curves of Nomogram Model Prediction in Different Cohorts (A-H)** The calibration curve of nomogram model prediction at 1-, 3- and 5- year predicted probability in TCGA_LIHC cohort (A-C), GSE14520 cohort (D-F), and 1- and 3- year predicted probability in ICGC_JP cohort (G-H).

**Supplementary Table S1 CCL18 signal pathway related genes were obtained from WikiPathways.**

| **CCL18 signaling pathway genes** | | |
| --- | --- | --- |
| ACTA2 | CCL3 | CDH5 |
| FGF2 | ITGA5 | LIMK1 |
| MTOR | PTEN | STAT3 |
| AKT1 | CCR1 | CFL1 |
| FN1 | ITGAL | LIN28A |
| NFKBIA | PTK2 | TGFB1 |
| ANXA2 | CCR2 | CHUK |
| FOS | ITGB1 | LIN28B |
| NRAS | PTK2B | TIMP1 |
| ARF6 | CCR3 | COL1A1 |
| FZD4 | ITGB2 | MAD1L1 |
| PIK3R1 | RAC1 | TIMP2 |
| ARNT | CCR4 | COL1A2 |
| GPER1 | ITPKB | MAPK1 |
| PIK3R3 | RELA | TWIST1 |
| ASAP1 | CCR5 | CREB1 |
| GSK3B | JAK2 | MAPK3 |
| PITPNM3 | SMAD2 | VCAM1 |
| ASAP3 | CCR6 | CXCL8 |
| IKBKB | KAT2B | MAPK8 |
| PLCG1 | SMAD3 | VEGFA |
| BMI1 | CCR8 | CXCR4 |
| IKBKG | KIF5B | MAPK9 |
| POU5F1 | SNAI1 | VEGFC |
| CASP8 | CDC25C | ELMO1 |
| IL10 | KITLG | MMP2 |
| PRKCA | SNAI2 | VIM |
| CCL18 | CDH1 | EP300 |
| IL15 | LDHA | MMP3 |
| PRKCD | SP1 | ZEB1 |
| CCL2 | CDH2 | EZR |
| IL6 | LDHB | MTDH |
| PRKCZ | SRC | ZEB2 |

**Supplementary Table S2 Sequences of primers used for pCDNA3.1(+) vector construction.**

| Primer | Sequence |
| --- | --- |
| CDC25C-FP  CDC25C-RP  v-CDC25C-FP  v-CDC25C-RP  CCR3-FP  CCR3-RP  v-CCR3-RP  v-CCR3-FP  RAC1-FP  RAC1-RP  v-RAC1-RP  v-RAC1-FP  LDHA-FP  LDHA-RP  v-LDHA-RP  v-LDHA-FP  CFL1-FP  CFL1-RP  v-CFL1-RP  v-CFL1-FP  BMI1-FP  BMI1-RP  v-BMI1-FP  v-BMI1-RP | ATGTCTACGGAACTCTTCTCATCCAC  TCATGGGCTCATGTCCTTCACC  TGAAGGACATGAGCCCATGAAACTAGAGAACCCACTGCTTACTG  GAGAAGAGTTCCGTAGACATAGCCAGAGAGCTCTGCTTATATAG  ATGACAACCTCACTAGATACAGTTGAGACC  CTAAAACACAATAGAGAGTTCCGGCTCTG  GTATCTAGTGAGGTTGTCATAGCCAGAGAGCTCTGCTTATATAG  AACTCTCTATTGTGTTTTAGAACTAGAGAACCCACTGCTTACTG  ATGCAGGCCATCAAGTGTGTG  TTACAACAGCAGGCATTTTCTCTTCCTC  ACACACTTGATGGCCTGCATAGCCAGAGAGCTCTGCTTATATAG  GAAAATGCCTGCTGTTGTAAAACTAGAGAACCCACTGCTTACTG  ATGGCAACTCTAAAGGATCAGCTG  TTAAAATTGCAGCTCCTTTTGGATCCCC  TGATCCTTTAGAGTTGCCATAGCCAGAGAGCTCTGCTTATATAG  AAAAGGAGCTGCAATTTTAAAACTAGAGAACCCACTGCTTACTG  ATGGCCTCCGGTGTGGCTGTCTC  TCACAAAGGCTTGCCCTCCAGGGAG  ACAGCCACACCGGAGGCCATAGCCAGAGAGCTCTGCTTATATAG  TGGAGGGCAAGCCTTTGTGAAACTAGAGAACCCACTGCTTACTG  ATGCATCGAACAACGAGAATCAAGATCAC  TCAACCAGAAGAAGTTGCTGATGACCC  CAGCAACTTCTTCTGGTTGAAACTAGAGAACCCACTGCTTACTG  ATTCTCGTTGTTCGATGCATAGCCAGAGAGCTCTGCTTATATAG |

**Supplementary Table S3 Sequences of primers used for qRT-PCR.**

| Primer | Sequence |
| --- | --- |
| qPCR-CCR3-FP  qPCR-CCR3-RP  qPCR-LDHA-FP  qPCR-LDHA-RP  qPCR-RAC1-FP  qPCR-RAC1-RP  qPCR-CDC25C-FP  qPCR-CDC25C-RP  qPCR-BMI1-FP  qPCR-BMI1-RP  qPCR-CFL1-FP  qPCR-CFL1-RP | GTCATCATGGCGGTGTTTTTC  CAGTGGGAGTAGGCGATCAC  ACGTGCATTCCCGATTCCTT  AACAGCACCAACCCCAACAA  TACGCCCCCTATCCTATCCG  CGCACCTCAGGATACCACTTT  ATGACAATGGAAACTTGGTGGAC  GGAGCGATATAGGCCACTTCTG  CCACCTGATGTGTGTGCTTTG  TTCAGTAGTGGTCTGGTCTTGT  TTCAACGACATGAAGGTGCGT  TCCTCCAGGATGATGTTCTTCT |
